# NOT ALL CREATED EQUAL: THE IMPACTS OF POSITIVE, NEGATIVE, AND PARTNER RELATIONSHIPS ON COGNITION IN LATELIFE

**DOI:** 10.1093/geroni/igad104.3710

**Published:** 2023-12-21

**Authors:** Nicole Ee, Fiona Matthews, Ruth Peters, Kaarin Anstey

**Affiliations:** University of New South Wales, Sydney, New South Wales, Australia; Newcastle University, Newcastle upon Tyne, England, United Kingdom; The George Institute for Global Health, Newtown, New South Wales, Australia; University of New South Wales, Sydney, New South Wales, Australia

## Abstract

**Background:**

Social engagement has been linked to higher cognitive function in later life and it is a well evidenced protective factor against dementia and cognitive decline. However, far less is understood about how the type, quality and quantity of social engagement differentially affects cognitive health outcomes in older adults.

**Methods:**

The sample consisted of 1972 participants aged 68-74 (M = 70.60, SD = 1.49) from a large population-based study in Australia, Personality and Total Health Through Life (PATH). The constructs underlying nine observed measures of social engagement were first identified through exploratory and confirmatory factor analysis (CFA). A Latent Variable Path Model (LVPA) was then employed to examine the relationships between positive, negative and partner social engagement on memory and executive function, as measured by six validated cognitive tests.

**Results:**

The CFA identified partner relationship as a distinct social engagement construct, separate to positive and negative relationships. The main analysis demonstrated that negative relationships and partner relationship were positively associated with better memory and executive function, with the former twice the strength of the latter.

**Discussion:**

These findings provide support that more frequent social interactions and larger support structures contribute to better cognitive function in later life, with partner relationship making a significant and distinct contribution. Positive or high-quality social engagement, however, appears to be a less critical factor in bolstering cognitive health. Close and cognitively demanding relationships may promote cognitive health via frequent cognitive stimulation, but more research is required to confirm the mechanisms underpinning observed effects.

